# Quantifying cerebral asymmetries for language in dextrals and adextrals with random-effects meta analysis

**DOI:** 10.3389/fpsyg.2014.01128

**Published:** 2014-11-04

**Authors:** David P. Carey, Leah T. Johnstone

**Affiliations:** Perception, Action and Memory Research Group, School of Psychology, Bangor UniversityBangor, UK

**Keywords:** cerebral asymmetries, language, handedness, WADA test, laterality

## Abstract

Speech and language-related functions tend to depend on the left hemisphere more than the right in most right-handed (dextral) participants. This relationship is less clear in non-right handed (adextral) people, resulting in surprisingly polarized opinion on whether or not they are as lateralized as right handers. The present analysis investigates this issue by largely ignoring methodological differences between the different neuroscientific approaches to language lateralization, as well as discrepancies in how dextral and adextral participants were recruited or defined. Here we evaluate the tendency for dextrals to be more left hemisphere dominant than adextrals, using random effects meta analyses. In spite of several limitations, including sample size (in the adextrals in particular), missing details on proportions of groups who show directional effects in many experiments, and so on, the different paradigms all point to proportionally increased left hemispheric dominance in the dextrals. These results are analyzed in light of the theoretical importance of these subtle differences for understanding the cognitive neuroscience of language, as well as the unusual asymmetry in most adextrals.

## General introduction

The curious relationship between language-related asymmetries in the human brain and handedness was a fundamental question for neuropsychological and behavioral neuroscience over almost all of the twentieth century. Sadly, this question has become of more specialist interest in the last 20 years or so, as paradigms in the cognitive neuroscience of language become increasing less focused on questions related to the left and the right cerebral hemispheres. In parallel, during the 1970s and 1980s handedness researchers gradually became embroiled in methodological arguments over issues such as preference vs. performance, the precise definition of adextrality, and how measures of hemispheric specialization interact with fashionable covariates such as handwriting posture, sex, familial sinistrality, and so on. Because the relationship between handedness and language laterality is subtle (e.g., Baynes and Long, 2007), statistical differences between right handers and non-right handers are not always obtained, leading some scientists to conclude that these groups are effectively homogeneous with respect to cerebral asymmetry. Many studies, (in functional magnetic resonance imaging [fMRI] in particular), have avoided any controversy (or, theoretically, unnecessary additional variance) by restricting analysis to dextrals (e.g., Berlin et al., 1973; Lindell and Nicholls, 2003; Voyer and Ingram, 2005; Hirnstein et al., 2010). In others, differences between dextrals and adextrals are **examined**, but the results are often inconclusive about real differences. Tzourio-Mazoyer et al. (2014) report some subtle anatomical differences between dextrals and adextrals, but find no functional activation contrasts in a large sample. Szaflarski et al. (2012) find that adextral children are 85% left brain dominant for language, hardly less than the dextral sample using their methods. Van der Hagen and colleagues argue that the effects of handedness on cerebral asymmetry are small, and suggest using direct measures of lateralization obtained from fMRI (see Van der Haegen et al., 2013b; also see Brysbaert, 1994).

This paper will attempt to reconcile evidence from several sources that all speak to the puzzling relationship between language dominance and dominant hand (particularly in the non-right handed, “adextral” population). In particular, we want to establish up to date estimates of any differences between dextrals and adextrals, comparing fMRI and other modern neuroscientific methods of the late twentieth and twenty-first centuries with the well-worn techniques of WADA testing, dichotic listening and visual half field experiments which dominated earlier in the twentieth century. To this end, we use meta analysis to try and establish whether there is a consistent difference between dextrals and adextrals, and if so, what is the best estimate for its magnitude, on average.

Several challenges are common across the diverse methods which examine language lateralization in individual people. Even within the specific paradigms outlined below, many studies differ in task, the reliability of the measurements, and the inclusion criteria for each group, in particular for the adextral sample. Within task, there is now some evidence to show that different strategies that individuals use can dramatically affect laterality quotients (for example, attentional biases/strategies may, and often do, muddy measurement of perceptual bias in dichotic listening experiments; Hugdahl and Andersson, 1986; Hugdahl et al., 2008; Hiscock and Kinsbourne, 2011). These direct attention blocks have also become very popular for experiments which help identify the relative contributions of bottom up and top down processes in auditory perception (Hirnstein et al., 2013; Passow et al., 2014).

Nevertheless, some of these concerns about between-study differences are less crucial if the data are treated meta-analytically. If some moderating variable, like sex or familial left handedness (Bishop, 1990), for example, is not balanced across two studies, differences in lateralization obtained may depend to an unknown degree on that unmeasured covariate. This sort of problem is lessened quite dramatically by an approach which produces a central tendency, rate ratio or effect size across many experiments (where presumably the distribution of the potential covariates will average out as simply another source of noise). In some sense, a multitude of studies which are heterogeneous, yet in spite of their differences, tend to point in the same direction when viewed collectively, is a strength rather than a weakness from a meta-analytic perspective.

Our working hypothesis for this set of studies is that all of the paradigms meta analyzed below will produce crudely equivalent group differences in proportions of left hemispheric speech/language dominance of approximately 15–20%, favoring the dextral samples. For researchers interested in handedness, such a result would come as no surprise (Willems et al., 2014). Other scientists are less concerned about differences between dextral and adextral people (as the majority of both groups share direction of cerebral dominance, on average) or argue that handedness and cerebral dominance may be confounded with one another (Hervé et al., 2013). Perspective of the researcher may be relevant here: for an electrophysiologist studying a language-related waveform, exclusion of adextrals may be unnecessary for two reasons: adextrals are rare, (typically representing roughly 10% of the population), and in any case, most of them will be lateralized in a fashion similar to dextrals. Nevertheless, for many neuropsychologists, the relationship between handedness and cerebral asymmetry is real and needs explaining. In some sense, it is ironic that interest in handedness and asymmetry has waned as newer techniques (that the neuropsychologists of the 1970s could not have dreamed of) have been developed. Our goal in this paper is to help establish the most accurate estimates, on average, for left hemispheric dominance in dextrals and adextrals, and to suggest why these proportions are important for providing fresh impetus to this field.

### General materials and methods

Each of the paradigms/domains described below were systematically searched for papers that included estimates related to speech and language asymmetry for samples of dextral and adextral participants. Potential studies were selected from computerized databases (ScienceDirect, PubMed) and Google Scholar searches, as well as from the reference lists of all papers collected previously which met the inclusion criteria. We also relied quite heavily on related reference and cited reference searches. A few sources published in non-English languages were perused by colleagues for the relevant frequency data. In order to be included in the analysis, each study must have included dextral and adextral participants, and provided frequency data for those two groups on the dependent measures(s) related to cerebral asymmetry for language.

A “proportion” approach to the meta analytic procedures has been adopted, using frequency data for dextrals and adextrals rather than the more typical effect size measurements of differences in central tendency. A few papers using this latter kind of meta analysis have been conducted previously (language laterality and handedness: Kim, 1994; sex differences in handedness: Papadatou-Pastou et al., 2008). Such endeavors are useful in trying to establish whether there is a significant difference between dextrals and adextrals (or men and women) on a particular measure, but estimating average effect sizes of this sort cannot be unambiguously converted to estimates of *incidences* in groups of interest. Of course, groups may differ quite dramatically on some measure of central tendency, but the means and variances associated with those differences cannot unambiguously reveal *how many* individuals in each group showed a particular effect. These sorts of issues are explored in much more detail in the growing literature on individual differences, which tends to be rather critical about psychology's obsession with central tendency; see Kanai and Rees (2011), Vogel and Awh (2008), for some of the discussion.

In the particular case of hemispheric specialization, larger average asymmetries favoring dextrals are assumed to be due to a small number of the adextrals in the sample who are lateralized in the opposite direction (i.e., to the other hemisphere). Reduced asymmetries in many or all of the individuals in an adextral sample would require a rather different interpretation. In fact, comparing different measures of a hypothetical underlying construct will be facilitated if the proportions of each group showing a typical pattern are reported. This approach may also circumvent some of the difficulties with test-retest reliabilities of some measures of language-related asymmetry such as dichotic listening (see General Discussion).

One particular difficulty in comparing the different studies summarized in the meta analyses of this paper is that many of them use quite distinct criteria for assigning individuals to operationally-defined dominance groups. For example, many (but not all) studies opt for a “bilateral” or “no difference” category when measured asymmetries between visual fields in tachistiscopic tasks, ears in dichotic listening tasks, or hemispheres in the case of fMRI, do not exceed a pre-specified threshold of lateralization (discussed in Jansen et al., 2006; Seghier, 2008). This problem is circumvented here by grouping together bilateral and no difference groups with those who display asymmetries favoring the right hemisphere on a language task^1^.

All meta analyses were performed using MetaXL, developed by Barendregt and colleagues, available as freeware from http://www.epigear.com/index_files/metaxl.html.

For all the analyses *a rate ratio* meta analysis was used (these are referred to as *risk* ratios in some studies, including Experiment 1 on aphasia incidence). These compare the proportion of people in one binomial category to those in the other, and compare this proportion in two separate groups. Statistically, odds ratios, rather than rate ratios, have more attractive mathematical properties for this kind of analysis, such as symmetry about “no difference” in an effect. However, for the frequency data for all the techniques described below, there was no theoretical reason to suspect any differences in the “other” direction—e.g., adextrals being more susceptible to aphasia after left lesions than dextrals; more adextrals with right ear advantages, etc. Finally, rate ratios are easier to interpret.

All available studies for each paradigm were subjected to a random effects meta analysis (using the variance estimators recommended by DerSimonian and Laird, 1986). These techniques allow for statistical estimates of central tendency (as effect sizes, means, rate ratios and so on) and variability to be made across a number of different studies which examine *similar* dependent and independent measures. Fixed effects models assume that each individual study is sampling *the same* underlying population effect and that *all* variance from study to study is measurement noise, sampling error, subtle differences in test administration and so on. Random effects do not assume that all of the underlying studies sample an identical population effect (Haddock et al., 1998; Borenstein et al., 2010; Cumming, 2012); hence there are sources of variation (say, aphasia as classified by different measures in samples tested at slightly different times after the neurological insult, etc.), which will not be identical from study to study. One limitation of random effects methods, however, is that studies with smaller sample sizes can contribute more to the overall effect estimate, as they contribute more to estimates of between study variability—in fixed effects models smaller variances result in larger weights). Nevertheless, the rate ratios from fixed and random effects models will be very similar when the heterogeneity is small, so we favor the more conservative approach of random effects.

For the subsequent paradigm-specific meta analyses, differences in precise tasks used, sex of group members, cut-off procedures, how adextrals were recruited, sampling bias and so on, make it quite clear that a random effects analysis is appropriate. Having said that, these studies are all attempting to estimate, directly or indirectly, differences in hemispheric specialization related to language processing. Studies too numerous to mention have identified the non-perfect relationships between these different techniques (and at times some rather limited test-retest reliabilities) so will not be discussed further (but see Bryden, 1982; Brysbaert, 1994; Binder, 2011 for further discussion of many of the most pertinent issues).

## Meta analysis 1: aphasia incidence after unilateral brain damage

### Introduction

Most of the early research which addressed language lateralization and handedness depended on studies of aphasic disturbances in individuals (Critchley, 1954). Early attempts to link *right* hemispheric language lateralization to left hand preference for writing (lumped together, erroneously, in fact, as “Broca's rule” e.g., Eling, 1984; Harris, 1991) were discredited quite quickly in the late nineteenth and early twentieth century (Harris, 1991). Case studies, too numerous to review here, have documented examples of “crossed” aphasia and apraxia in single individuals (e.g., aphasia or apraxia after a right hemisphere lesion in a right hander or, much more rarely described, the left hemisphere in a left hander; reviewed in Alexander and Annett, 1996; Coppens et al., 2002). As important as these single cases studies were, it was the large sample studies of aphasia and handedness that debunked the idea of a perfect link between hand preference and a speech-dominant contralateral hemisphere.

These rather laborious group studies of hospital patients with and without aphasia were the first datasets that suggested that right hemispheric dominance in adextrals was not the norm by any stretch of the imagination. Unfortunately, the accuracy of estimates from this research is complicated by the anti-sinistral biases that were common, even in Western cultures, for anyone born prior to World War 2. Inevitably, some proportion of left-handed writers will have been forced to switch their handedness at an early age. Similarly, “left handers” in such cohorts were probably those individuals most resistant to direct or indirect pressures to switch to the preferred right hand (this topic is reviewed in Siebner et al., 2002; Searleman and Porac, 2003) Considering the average age of many stroke patients, these sources of bias will have had persisting effects well into the twentieth century. Unfortunately, dissipation of such effects is happening too late, as such large sample studies have become more expensive and less fashionable. In some of the early experiments, heroic efforts were made to document cases of handedness switch in some “right” handers (e.g., Gloning, 1977), but these were the exception rather than the norm.

For inclusion in this analysis, three criteria needed to be met. First, aphasia incidence needed to be estimated in groups of dextrals and adextrals using the same tests and criteria. Second, the number or proportion of dextrals and adextrals who were so diagnosed out of larger samples of unilateral brain damage was reported. Finally, we expected no admission or strong suggestion of pre-selection of non-right handers in any way that would bias estimates of aphasia frequency after left or right brain damage (see below).

### Methods

Literature searches in Pubmed revealed 1100 sources when “aphasia” and “handedness” were searched for (September, 2014). Many of these studies: (1) only provide the mean handedness of an exclusively dextral sample; (2) are single case reports; and (3) compare treatments of right and left handed dysphasics. Additional potential studies were sourced by cited reference searches of early papers on aphasia in adextrals including Basso et al. (1989, 1990), Brain (1945), Critchley (1954), Goodglass and Quadfasel (1954), Humphrey and Zangwill (1952), Zangwill (1960).

Unfortunately, several large scale studies of aphasia incidence do not report handedness (e.g., Laska et al., 2001; Chilosi et al., 2005) or do not report their data by side of lesion and handedness, as well as by presence or absence of aphasia (e.g., Bingley, 1958; Zangwill, 1960; Brown and Hécaen, 1976; Hécaen, 1976; Vargha-Khadem et al., 1985; Basso et al., 1990; Pedersen et al., 1995, 2004; Basso and Rusconi, 1998; Godefroy et al., 2002). For example they may report how many aphasics in a subgroup were right or left handed, but these sorts of data are not sufficient, without information about how many patients with left or right unilateral lesions *were not aphasic*. An additional restriction in this literature is that it has gradually shifted into looking for functional evidence for compensation in dysphasics within a damaged hemisphere or in the contralateral (presumably innately non-dominant) hemisphere. These newer studies often have small samples, and examine right-handed patients only which almost inevitably means lesions of the left hemisphere (e.g., Pettit and Noll, 1979; Heiss et al., 1999; Duffau et al., 2003; Krieg et al., 2013).

An additional methodological concern (as if there aren't enough already) in studies of aphasia incidence and handedness is that the earliest (and best cited) papers are not, in fact, composed of samples of *unselected* dextrals and adextrals with unilateral brain damage. Instead they are typically “compilations of scattered individual cases” (Kimura, 1983), where non-right handers were particularly noteworthy when they presented *with* aphasia, and *not so noteworthy when they did not*. For this reason one of the best known studies (Goodglass and Quadfasel, 1954) has been excluded from the analyses. In other experiments, inclusion criteria for individual patients included “adequate tests for aphasia” (Humphrey and Zangwill, 1952), which as Kimura (1983) notes, implies that some dextral and or adextral patients were not routinely tested. We have excluded this source as well.

Studies of unselected series of left and right brain damaged patients, which also recorded handedness are remarkably rare. As Kimura (1983) reports in one of the most cogent analyses, some selectivity (not necessarily described in the manuscript or book) of adextral cases is the norm rather than the exception (see also Annett, 1975, 2002). Nevertheless, we managed to identify 14 such studies, which are the subject matter of the first two meta analyses.

### Results

**Figure 1:**
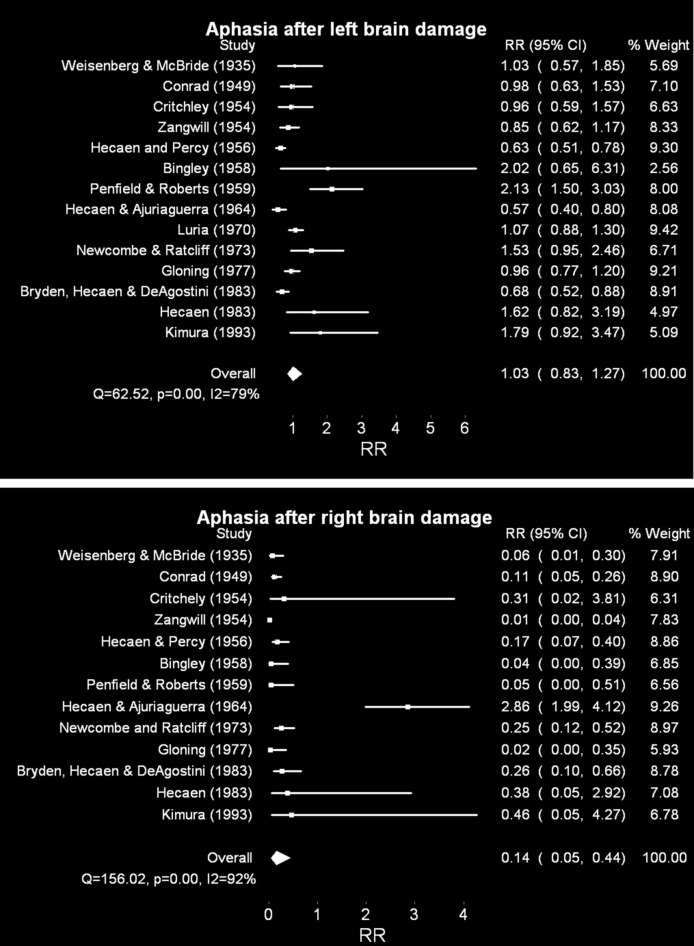
**Random effects meta analyses of relative risk of aphasia after unilateral brain damage, dextrals compared to adextrals**. Risk ratios greater than one suggest greater susceptibility of dextrals than adextrals; less than one greater susceptibility of adextrals than dextrals. *CI* = 95% confidence intervals. I^2^ is a measure of the percentage of total variation due to variation between studies. Note that no estimates of susceptibility were provided in Luria (1970) for right hemisphere lesions. **Top panel**: unilateral left brain damage. **Bottom panel**: unilateral right brain damage. For additional comments and the raw frequencies, for all figures, see Supplementary Materials.

The 14 studies of patients with left brain damage summarized included 2421 dextral and 390 adextral patients; the 13 studies^2^ of patients with right brain damage summarized included 1907 dextral and 256 adextral patients. The results of this analysis on aphasia incidence are plotted in Figure 1. (Supplementary Material contain the excel spreadsheets for this analysis, which provide the raw frequencies for dextrals and adextrals, the weights of each study in the final rate ratio estimate, and so on). In the top panel, the effects of unilateral *left* hemisphere lesions are depicted, comparing risk ratios calculated for dextral and adextral patients (in that order). A risk ratio in this context contrasts the number of unilateral brain damaged patients with aphasia to those without aphasia; this proportion in dextrals serves as the numerator to the same proportion in adextrals (therefore risk ratios greater than one indicate greater sensitivity in dextrals).

The associated Q statistic (62.52, *p* < 0.001) for aphasia after left brain damage suggests considerable heterogeneity across studies (which validates the use of a random, rather than a fixed-effects analytical strategy). I^2^, another measure, provides the percentage of total variance due to variation between studies. The pooled risk ratio suggests that there are no differences between dextrals and adextrals in terms of their susceptibility to aphasia after unilateral left hemisphere lesions: risk of 1.03 (95% C.I. = 0.83–1.27). These same data were heterogeneous across study (*I*^2^ = 79.21).

By contrast, the pooled risk ratio following right hemisphere lesions suggests a clear difference (bottom panel), although a considerably noisy one: odds of 0.15 (95% C.I. 0.05–0.44; *I*^2^ = 92.31) were obtained one. Stated in terms of *adextral relative to dextral* susceptibility, the risk ratio is 6.7 for adextrals to become dysphasic after a right hemisphere lesion relative to the dextral population. As with the left brain damage analysis above, perhaps unsurprisingly, heterogeneity of these estimates is large: *Q* = 156.02, *p* < 0.001. Many studies not included in the meta analysis quantify aphasia incidence in dextrals after left or right hemispheric damage. These studies result in a similar bias toward greater aphasia incidence after left hemisphere lesions (e.g., McGlone, 1977; Wade et al., 1986).

### Discussion

The noisiness of both of these overall effects is partly due to the sample sizes available, for the adextral patients in particular (e.g., adextral n's range for left lesions from 6 to 87; Supplementary Material; for right lesions from 2 to 53; Supplementary Material). Annett (1975, 2002) argues that the series also have different *proportions* of left handers, which shows different inclusion criteria, which will lead to different distributions of speech lateralization. In spite of this heterogeneity, these data suggest that dextrals and adextrals are similarly prone to aphasia after left hemisphere lesions, and that right hemisphere lesions are much more likely to produce dysphasia of some sort in adextrals compared to dextrals. It is tempting to relate the similarity of the two handedness groups in susceptibility to dysphasia after left lesions to statistical noise plus the considerable evidence suggesting that adextrals are *largely* left brained for language, as are the dextrals, of course. Nevertheless, the lack of even a small difference favoring increased incidence in the dextrals is puzzling. Sample size clearly is at issue here, but the samples for the second meta analysis (patients with right brain damage) are similarly limited. The absence of aphasic symptoms in people with left hemisphere damage might mean that they don't present to neurologists or stroke specialists who compile some of these group studies (Levy, 1974; Annett, 2002)—perhaps more of an issue for adextrals after left hemisphere lesions (relative to dextrals), but such selectivity could also affect incidence estimates for aphasia in dextrals after right lesions.

Nevertheless, this meta analysis does suggest that dextrals and adextrals do not differ in terms of susceptibility to aphasia after left hemispheric lesion. Few of the investigators of the original studies have commented on this particular *symmetry*. In part, the absence of a difference in many of the papers was interpreted in terms of *refuting* “Broca's rule”—what was noteworthy at the time was that the adextrals *were not right brained* for language. Comment on their striking similarity to dextrals is infrequent.

As an aside, some of these early aphasia studies also suggested two parallel but slightly counterintuitive hypotheses about aphasic syndromes in non-right handers. First, Chesher (1936) and Gloning et al. (1969) provided early evidence to show that adextrals were more likely to have dysphasia after brain damage than dextrals (see also Hécaen and Percy, 1956; Satz et al., 1965; Satz, 1979; although Newcombe and Ratcliff, 1973; Kimura, 1983; contest these claims). Second, comparing prognosis in right and non-right handers after becoming dysphasic, the adextrals tend to recover somewhat earlier and more completely (Subirana, 1969; although this claim is also contentious, see Pedersen et al., 1995). These somewhat contradictory findings are in part more understandable (at least) when data from large scale studies of another group of patients began appearing in the 1960s and ideas of *bilateral speech* representation, in adextrals at least, became more commonly understood. It is also tempting to relate the first claim to the current findings; similar dysphasia risk after left lesions but increased risk after right lesions.

## Meta analysis 2: WADA testing in pre-surgical epileptic patients

### Introduction

Another class of neuropsychological data, distinct from the laborious large sample aphasia incidence studies, has come to dominate thinking about language lateralization and handedness (and unlike the aphasia studies, such experiments continue to be performed to this day). Juhn Wada popularized a technique for determining language lateralisation in pre-surgical candidates for epilepsy surgery (Snyder and Harris, 1997; Wada, 1997). A great advantage of anesthetizing each hemisphere in turn and testing for speech arrest is that participants could be classified trichotomously (left hemisphere dominant; right hemisphere dominant, or bilateral). Bilateral classification was a consequence of either no speech arrest after amytal to either hemisphere (a type one of us refers to students as “good bilateral”) or speech arrest after amytal to either hemisphere (“bad bilateral”). Some researchers claim that speech arrest in these later cases can be somewhat less severe than what is typically obtained from patients with epilepsy with more straightforward unilateral speech dominance.

In any case, this technique, in the capable hands of Milner, Rasmussen, Penfield and others at the Montreal Neurological Institute, led to the most popular estimates of speech lateralization in dextrals and adextrals (see Table 1). This popularity is somewhat surprising, as of course, most people with intractable epilepsy would have had brains that had dealt with congenital abnormalities for a lifetime; perhaps not the most representative sample for asymmetry estimation in the neurologically-intact brain^3^.

**Table 1:** **Trichotomous classification of speech and language dominance in 266 epileptic patients using the WADA test (Rasmussen and Milner, 1977)**.

	**Speech lateralization**		
	**Left hemisphere (%)**	**Bilateral (%)**	**Right hemisphere (%)**
**GROUP**			
Dextrals	96	0	4
Adextrals	70	15	15

Since these early observations, large scale studies using the WADA test have been published on several occasions. The availability of these large n datasets is made possible by the tremendous popularity of the technique in neurosurgery units, even after fMRI availability had become widespread (Baxendale et al., 2008; Wagner et al., 2012). These studies, often with more refined techniques and definitions, are subject to the caveat of potentially abnormal hemispheric lateralization in people with congenital brain abnormalities (Kimura, 1993; Annett, 2002). Nevertheless, the estimates for the most part are largely consistent with the Rasmussen and Milner percentages presented in Table 1. Literature searches revealed 350 (partially overlapping) sources when (“WADA” or “IAT” or “sodium amytal”) and “handedness” were searched for in PubMed (September, 2014). Additional potential studies were sourced by cited reference searches of early papers on WADA and handedness including Binder et al. (1996), Rasmussen and Milner (1977), Woods et al. (1988) or came up in our other PubMed and Google scholar searches.

### Results

**Figure 2:**
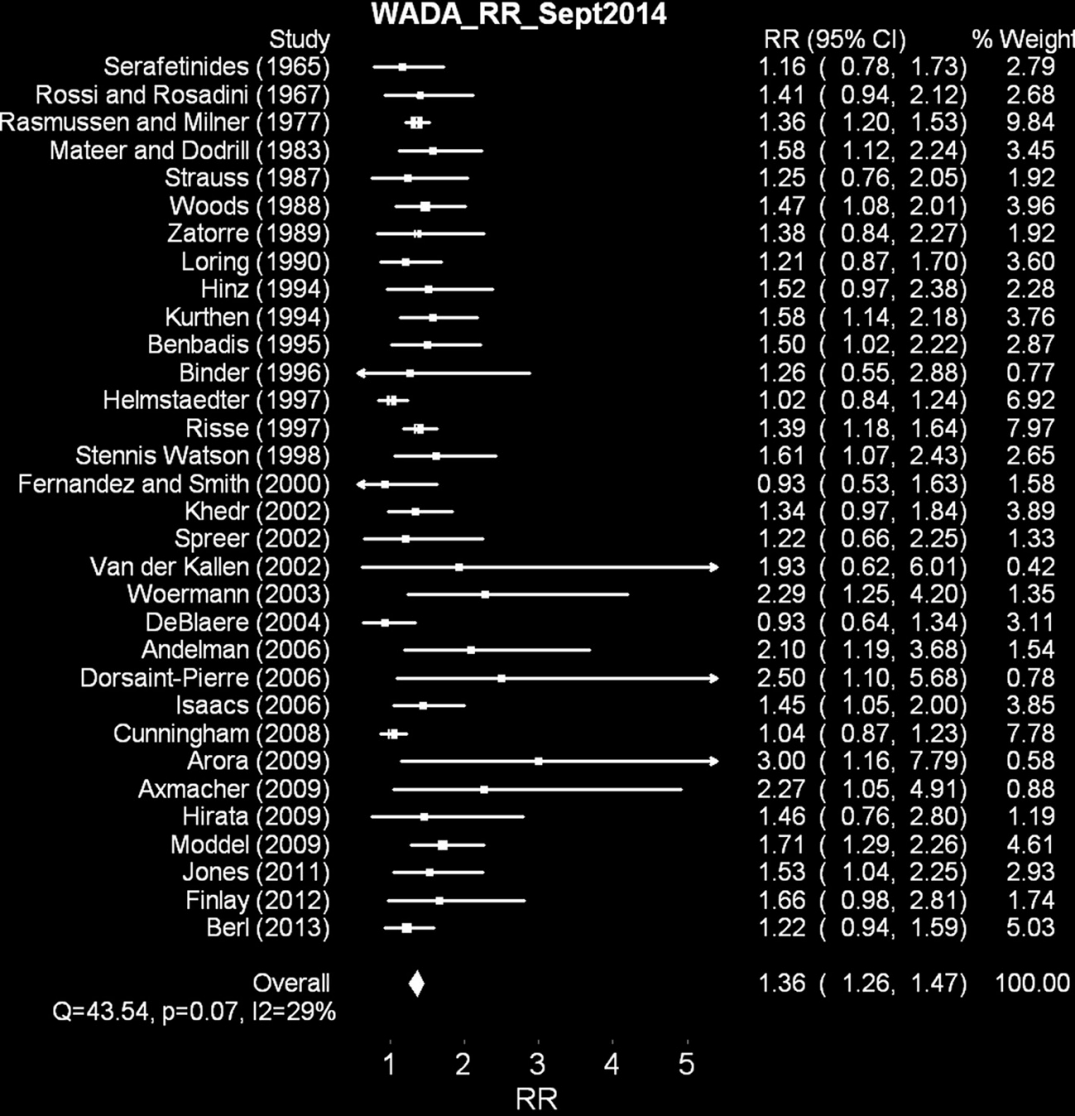
**Random effects meta analysis of WADA test left brain dominance relative to anomalous dominance for dextrals relative to adextrals**. Note that the range of the 95% confidence intervals for the overall effect, does not overlap zero.

**Figure 3:**
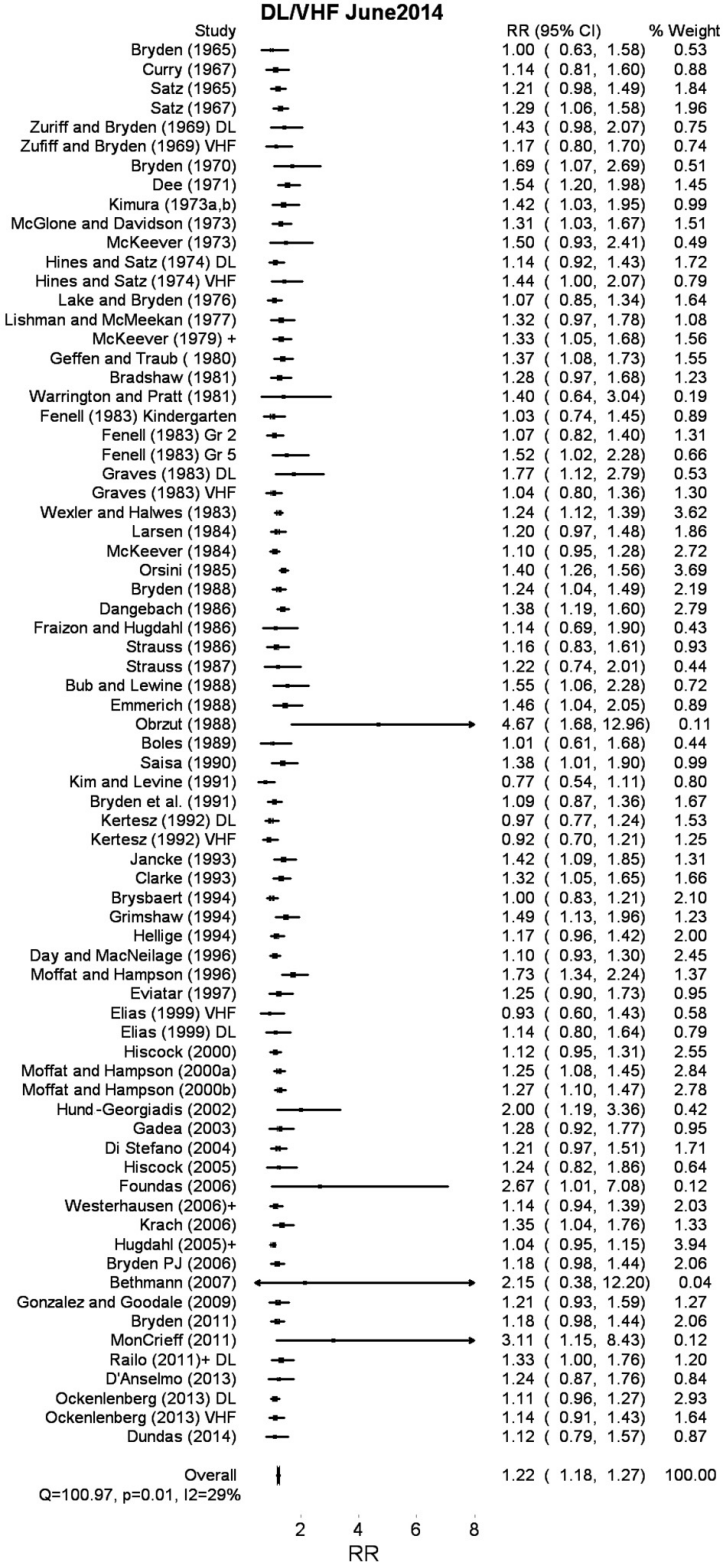
**Random effects meta analysis of right ear/right visual field bias for dextrals relative to adextrals**.

**Figure 4:**
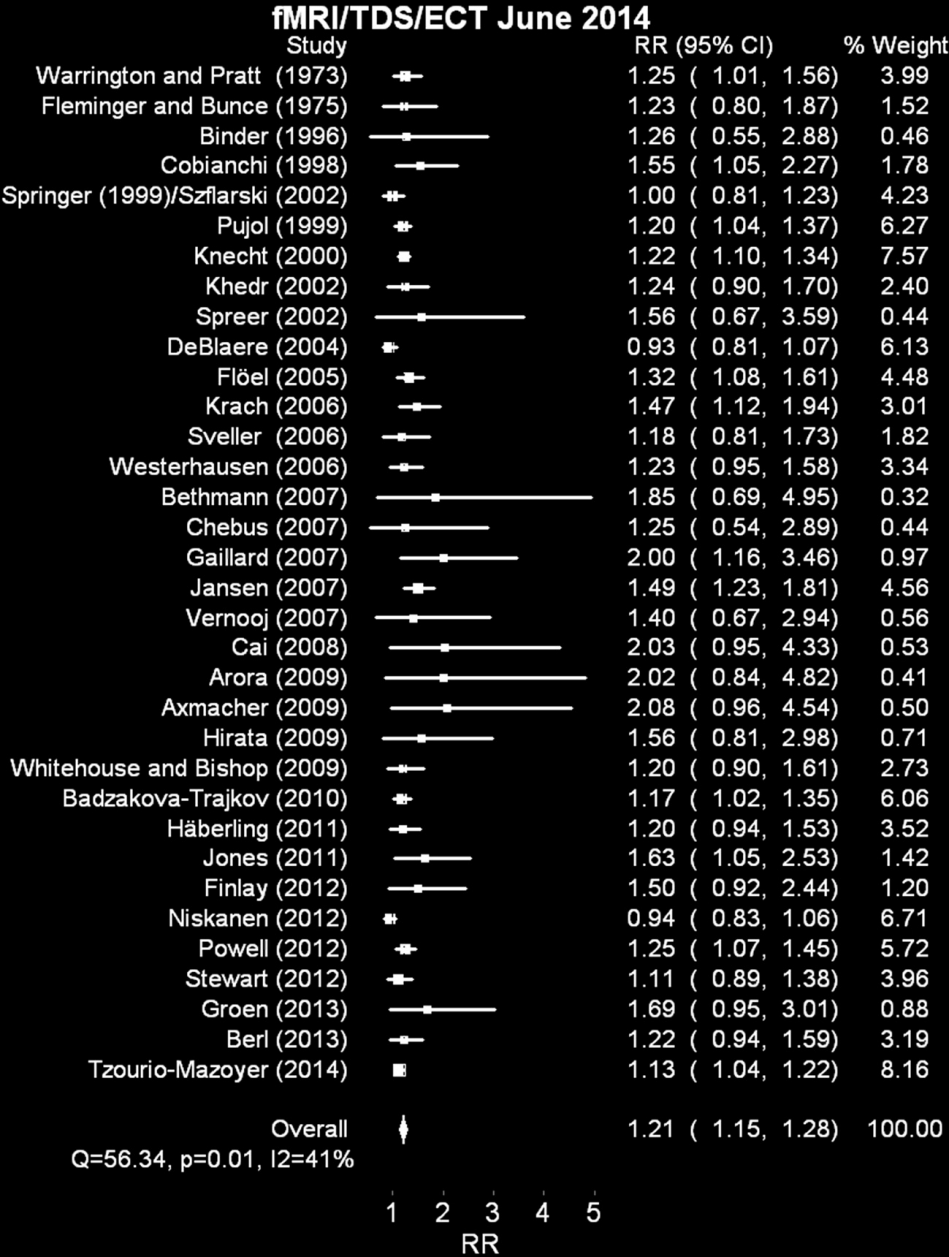
**Random effects meta analyses of fMRI + rate ratio of left brain dominance to anomalous dominance in dextrals relative to adextrals**. The analysis including the excluded study (Basic et al., 2004) is available as Supplementary Material.

The 32 studies summarized included 2771 dextral and 738 adextral patients. The results of the random effects meta analysis of these studies appears in Figure 2. Supplementary Material contains the associated Excel file with the raw data, weights for each study and a description on a separate sheet of some of the studies checked but not included in the analysis. In this comparison, unlike in the aphasia incidence meta analyses above, dextrals and adextrals are compared in one analysis, which contrasts the risk ratios (in this case some investigators would refer to it as a rate ratio) of *left brain dominance* relative to *anomalous dominance* for speech. In this latter category, in the studies where bilateral dominance was occasionally assigned, these cases were pooled with right brain dominance (this convention is also followed in Figures 3, 4 for the dichotic listening [DL]/visual half field [VHF] data and the fMRI/ECT/TDS data, respectively).

These data, collectively, contain less heterogeneity across studies (*Q* = 43.54, *p* < 0.07 NS; *I*^2^ = 28.81%) than the aphasia meta analyses above. The rate ratios for 30/32 studies are greater than 1 (range 0.93–3.00). The overall rate ratio is 1.36 (95% CI 1.26–1.46). These data suggest that left brain dominance, at least in epileptic patients selected for and assessed using the WADA test, is more common in dextral populations than adextral ones.

We also modeled % left dominance in the two groups by weighting each group percentage (n left dominant/n left dominant + n anomalous dominance ^*^ 100) by the meta-analytically derived inverse weighting (See “weighted means” columns in Supplementary Materials). This procedure suggests a best estimate, on average, of left brain dominance in dextrals of 87% and in adextrals, 65%). The former proportion is not radically different from other studies that report WADA data from large dextral samples only (e.g., Benbadis et al., 1995b; see Ocklenburg et al., 2014 for a recent review; also see more rare adextral-only studies such as Perlaki et al., 2013).

### Discussion

These results point to increased left hemisphere dominance in dextrals relative to adextrals, in contrast with the left brain damage aphasia incidence data reported in Meta Analysis 1. Of course, there are many scientific caveats related to these samples, most of which are distinct from those related to the aphasia incidence data analyzed above. For example, there is some debate about how to classify individuals as bilateral for speech (surprising, in that a binary speech arrest yes/no classification should be possible after WADA testing; for reviews see Snyder et al., 1990; Baxendale, 2002). For example, Benbadis et al. (1995a) contrast this “arrest, yes or no” kind of approach with duration of speech arrest and relative speech criteria (i.e., L-R/L+R, a type of normalizing laterality quotient much more common in the dichotic listening literature mentioned below). When they adopted 2/3 of these measures to indicate bilateral representation, they could group these patients into bilateral autonomous (either hemisphere on its own can support speech) and bilateral dependent (both hemispheres show equivalent speech arrest). This proportion was roughly 50:50 (but only 19/165 patients achieved classification as bilateral with this set of criteria). They did not find much of a difference between the proportions of dextrals and adextrals in either bilateral or right dominance groups.

The current results suggest more dramatic differences between dextrals and adextrals (in the case of the left hemisphere in particular) than in the aphasia incidence literature summarized in Figure 1. One problem for estimating hemispheric asymmetry in non-epileptics from these studies is that, of course, many people suffering from severe epilepsy will have had congenital abnormalities, which may in some instances at least, lead to a change in speech and language dominance from one hemisphere to another. Of course, this caveat *should* apply to the estimates of speech dominance in the dextrals as well as the adextrals (assuming, probably wisely, that epileptogenic foci are rather agnostic about which hemisphere they choose to appear in), unless of course a more complicated “pathological left handedness” argument is made (e.g., Geschwind and Galaburda, 1985a,b,c). Claims for increased incidence of adextrality in epilepsy are extremely common. For example, Kim et al. (2001) claim a 15% incidence of left handedness in a sample of Korean epileptic patients with left temporal lobe epilepsy (TLE), compared to a control sample estimate of around 5% (a three-fold increase); strong right handers were more common, but less dramatically so, in a right TLE group (84%) compared to the strong right hander incidence in the control group (67%). However, this sample was selected for medial temporal lobe epilepsy exclusively (and not for testing handedness incidence in epileptic people *per se*), as well as for relatively equal numbers of left TLE and right TLE patients (58 and 51, respectively). In another series, this time of 92 consecutive epileptic people with left hemisphere foci, 71 were right handed (79%), and 20 were left handed or ambidextrous (21%); the comparable numbers with right hemisphere foci were 55 right handers (77%) and 16 adextrals (23%; Stewart et al., 2014). These numbers can be compared to normal incidences in post-World War II Western societies of about 90% dextral, 10% adextal (McManus, 2002). Slezicki et al. (2009) find an increased incidence of adextrality of approximately 6% in American and 3% in Korean samples, totaling 478 people with epilepsy. Dellatolas et al. (1993) do find an increased incidence of left handedness in people with epilepsy, but only significantly so in individuals with brain damage so severe that they were hemiparetic. These data suggest that in some, but not all, series of epileptic patients, adextrality may be slightly more common that is usually detected in non-epileptic samples.

In fact, a little known consideration suggests that the dextrals in the original Montreal Neurological Institute cohort are not a completely random sample of all right-handed epilepsy patients. Reading the fine print of Rasmussen and Milner (1977) reveals that many dextral epileptic patients were *not* routinely given the WADA test prior to their surgeries; for example, those without adextral family members^4^. (Presumably left brain dominance was assumed in these dextral surgical candidates). Of course, if they weren't screened, they could not have contributed to the estimates in Table 1. Adextrals, as a matter of course, were tested regularly (also see Rey et al., 1988; Knake et al., 2003, for similar inclusion restrictions in other neurosurgical settings). One would think that the estimates for pre-selected dextrals could be “watered down” with respect to a “true” left hemispheric dominance measure, available only if *all* right handers were routinely administered the WADA. We think that this is unlikely, given near ceiling estimates of left dominance from most WADA studies (which is also consistent with much of the evidence in non-epileptics below), but keep the “pre-selection” issue in mind when reading critiques of WADA as a legitimate estimator of language lateralization in adextrals.

This issue of reorganization after early right or left hemisphere damage has been addressed to some extent by studies such as Stewart et al. (2014), Cunningham et al. (2008), Powell et al. (1987) and others, who identify speech dominance in patients with epileptic foci in the right hemisphere or the left. Eight of these studies are summarized in Table 2 (sadly, these include four experiments, of many, where only left temporal lobe epileptic patients were included). Samples with small n's (e.g., Staudt et al., 2002), no report of handedness or dextrals only (e.g., Helmstaedter, 1999; Brázdil et al., 2003; Raja Beharelle et al., 2010) or studies which have utilized pre-selection to include more people with anomalous dominance (e.g., Strauss and Wada, 1983) are omitted. Obviously studies that report the number of dextrals and adextrals in the sample, but do not provide separate language dominance for each group as a function of hemispheric locus, are also not included. A weighted means analysis does support the idea that left lesions (relative to right lesions) do drive up the incidence of anomalous dominance in the adextrals by over 26% (although note the tiny sample sizes) while in dextrals the increase is much smaller (about 11%).

**Table 2:** **Speech dominance as a function of handedness and side of lesion/epileptic focus**.

	**Left hemisphere lesions**				**Right hemisphere lesions**			
	**Adextrals**		**Dextrals**		**Adextrals**		**Dextrals**	
	**LD**	**AD**	**LD**	**AD**	**LD**	**AD**	**LD**	**AD**
Cunningham et al., 2008	41% (7)	59% (10)	84% (53)	16% (10)	75% (9)	25% (3)	100% (55)	0% (0)
Gaillard et al., 2007	40% (8)	60% (12)	78% (64)	18% (22)	–	–	–	–
Helmstaedter, 1999	89% (16)	11% (2)	86% (4)	14% (9)	58% (7)	42% (5)	78% (58)	22% (16)
Mbwana et al., 2009	25% (2)	75% (8)	62% (23)	38% (14)	–	–	–	–
Powell et al., 1987	40% (2)	60% (3)	44% (4)	56% (5)	100% (3)	0% (0)	70% (7)	30% (3)
Stewart et al., 2014	55% (11)	45% (9)	85% (60)	15% (11)	94% (15)	6% (1)	93% (51)	7% (4)
Strauss et al., 1990	11% (1)	89% (8)	72% (13)	28% (6)	–	–	–	–
Strauss and Wada, 1983	25% (2)	75% (6)	94% (21)	6% (4)	100% (3)	0% (0)	100% (31)	0% (0)
Sveller et al., 2006	59% (13)	41% (9)	82% (50)	18% (11)	–	–	–	–
Weighted mean	58%	42%	80%	20%	84%	16%	91%	9%

*Strauss et al. (1990) do not indicate if any patients overlap with Strauss and Wada (1983) so both are included. Weighted means were calculated as a function of the total number of adextrals or dextrals in the LD and AD columns, separately for left and right hemispheric lesions*.

For expediency's sake, another crucial moderator of these effects has been ignored: whether or not the unilateral brain damage occurs early or late in life (the sample sizes in this domain, as usual in the adextrals in particular, do not inspire confidence in the parsimony of quantifying a four-way interaction between handedness group, language dominance, side of focus and age of injury).

In any case, anomalous dominance cannot be completely explained by left hemisphere damage in adextrals. Thirty years of functional and structural neuroimaging alone has put paid to any sort of “all left handedness (and/or anomalous dominance) follows from left hemisphere pathology,” pushing a few unfortunate people away from a near 100% right-handed, left-hemisphere dominant phenotype. In fact, we often forget that 5–10% dextrals, by most estimates (see Table 2), may have anomalous dominance. Few would argue that these individuals have left hemisphere pathology. The pathological left hander account cannot be dealt with in any detail here. It, in any form, is complicated by the fact that genetic models have yet to account for any causal direction of language dominance-handedness relationships. In other words, any innate plan could be for handedness, which drives, incompletely, speech and language dominance, or, could be for speech and language dominance which drives, incompletely, handedness (see McManus, 1985, 2002; Yeo and Gangstead, 1993; Corballis, 1997; Annett, 2002; Klar, 2003; Armour et al., 2013; for further discussion).

## Meta analysis 3: indirect techniques with neurologically-intact participants

### Introduction

WADA testing of the sort described above became quite a common exercise in neurosurgery clinics from the 1970s onwards; in parallel, experimental psychologists were pursuing less direct methods for examining behavioral asymmetries that are related (in theory) to cerebral asymmetry for language. The two main methods, dichotic listening (where different sounds are presented to the two ears simultaneously) and tachistiscopic studies (visual half fields, presenting stimuli such as words or consonant-vowel-consonant syllables) can provide sensible estimates of cerebral asymmetries that are largely consistent with the aphasia and WADA test research. Unfortunately, the tendency to provide the proportions of any sample of dextral or adextral participants who show, for example right ear or left ear bias, fell out of favor relative to the usual null hypothesis significance tests, contrasting groups defined by handedness (and occasionally, sex, writing posture, familial presence of adextrality, and so on). Inevitably, these rather laborious large n studies began to fall out of favor, partly due to the fact that the results showed that dextrals were more lateralized than adextrals on any particular indirect measure (see Bryden, 1982 for a comprehensive review of the relevant literature from 1960s to the 1980s)^5^.

In other studies, particularly ones with smaller sample sizes, mean differences between dextrals and adextrals on any particular dependent measure were not statistically significant, leading authors to conclude that handedness has no effect (e.g., Goodglass and Barton, 1963; Hugdahl et al., 2012) or more recently, that any effects of handedness are small relative to larger effects of more direct measures of language dominance (Van der Haegen et al., 2013b). Remarkably (to us, at least) Kimura herself, who helped launch dichotic listening as a valid paradigm in asymmetry research, had argued from some of her earliest data that dichotic listening scores do not discriminate between dextrals and adextrals (Kimura, 1961). Some years later, Bryden et al. (1983a), argued that hemispheric dominance accounts for about twice as much of the variance in dichotic listening as handedness does.

Nevertheless, these techniques might play some small role in identifying the probable language lateralization of individual people (if, for example, peripheral hearing differences between ears and attentional biases can be ruled out using forced attentional conditions, hearing tests and so on). They may also speak to estimates of the degree of left brain dominance in dextrals and adextrals if several weaker effects can be pooled using the techniques of meta analysis. Therefore, data on proportions of dextrals and adextrals who showed ear or visual field advantages were gleaned from the literature. Kim (1994) has previously performed an early meta analysis on VHF data; however his focus was on variance/central tendency in dextral and adextral groups rather than the proportions of participants in left right or bilateral language dominance categories.

### Methods

To be included in the current meta analysis studies must have provided frequencies of ear advantages (or visual field advantages) in dextrals and adextrals. These are, for historical reasons, more common in dichotic listening studies and much less common in divided visual field experiments (Hugdahl and Franzon, 1985). It may not be surprising to the reader by this point to learn that that many studies (sadly some with remarkably large samples) do not provide these data, and instead rely on inferential statistics on means and variances, test-retest correlations and the like (e.g., Orbach, 1967; Higenbottam, 1973; Briggs and Nebes, 1976; McKeever and VanDeventer, 1977; Hines et al., 1980; Geffen and Caudrey, 1981; Bryden et al., 1983b; Foundas et al., 2006).

### Results

The 73 studies summarized included 6691 dextral and 3497 adextral participants. The results of this random effects meta analysis appear as Figure 3. Supplementary Material contains the raw data, and the meta analytic weights associated with each study. It also contains a number of studies not included in the analysis on a separate sheet. The obtained odds ratio of 1.22 (95% CI 1.18–1.27) suggests that dextrals are more likely to show right ear/right visual field advantages relative to adextrals, in spite of considerable heterogeneity again (*Q* = 100.97, *p* = 0.01; *I*^2^ = 29.69%). Of the 73 studies, all but 4 result in rate ratios greater than 1. It may be worth noting that the vast majority of these participants would have been taken from samples of university undergraduates (a population, especially in the selective days of the twentieth century university sector, who would be unlikely to be overly populated by adextrals with subtle left hemisphere pathologies). In Supplementary Material, we have also multiplied each proportion for dextrals and adextrals by the weights assigned by the meta analysis. For dextrals, the weighted mean is 83.2% left hemisphere biased; for adextrals the weighted mean is 68.2%.

### Discussion

The direction of these data is consistent with the results of the WADA test analysis above, albeit with a slightly reduced pooled rate ratio in this case (1.22 vs. 1.37). It is difficult to unambiguously interpret this smaller rate ratio (in comparison with the WADA rate ratio reported above) as an effect of reduced sensitivity of indirect tests like DL and VHF experiments. Theoretically, measures that are more indirect would result in higher proportions of participants being assigned to the anomalous dominance category, but we can see no obvious reason why such a bias would interact in some meaningful way with handedness group. The final paradigm-driven meta analysis below may speak to this difference to some extent.

## Meta analysis 4: functional magnetic resonance imaging (fMRI), electro-convulsive therapy (ECT) and transcranial doppler sonography (TDS)

### Introduction

Other techniques were brought to bear in the 1970s which speak to language lateralization in dextrals and adextrals beyond the indirect perceptual techniques summarized above in Meta Analysis 4. For example, Elizabeth Warrington and Richard Pratt realized that inferences similar to those made using the WADA test could be made by studying speech arrest in patients undergoing electroconvulsive therapy (ECT) for psychiatric disturbances such as depression. In a sample of 55 right handed patients, speech dysfunction was elicited after left skull ECT in 100% of them (Pratt and Warrington, 1972). In a later study, Warrington and Pratt (1973) extended the method to 24 left handers and found left sided speech arrest in 70% of the sample. A later independent study by Geffen et al. (1978) reported 80% left hemispheric dominance in a sample of 31 right handed patients a few years later.

We have grouped ECT, used in this way, with the more modern methods described below as the similarity to transcranial magnetic stimulation (TMS) is a striking one. Of course, by the 1990s, additional technologies have been brought to bear on questions related to language laterality and handedness. Unfortunately (for our purposes here) many of the samples have largely been devoted to documenting the usefulness of fMRI as a replacement for the WADA test (see Medina et al., 2007; Bauer et al., 2014 for reviews of this extensive literature), typically in smaller samples of patients about to undergo epilepsy surgery. We use the term smaller here regarding our purposes of course, which in an ideal world would include many dextrals and adextrals reported on as separate groups. Understandably, these small samples tend to contain very few (if any) adextral participants (Desmond et al., 1995; Binder et al., 1996; Worthington et al., 1997). A similar problem exists for several papers which have attempted to use repetitive TMS for the same purpose (Abou-Khalil, 2007, reviews several of these papers). The exception to this rule is included (Khedr et al., 2002).

Nevertheless, a handful of fMRI—WADA comparison experiments (and a small number of papers using other methods, such as TDS and magnetoencephalography—MEG) have collected either so many participants over time that a number of adextrals are included, or, rarely, have by design pursued additional adextrals (usually to increase likely variance in speech dominance). Thirty-five such studies, as well as large n fMRI studies in non-epileptics which include adextrals, are summarized in Table 3 ^6^ ^7^. Note that many of these experiments will be based on epileptic participants, so will be subject to the same caveats mentioned above regarding WADA study results.

**Table 3:** **fMRI/ECT/TDS studies of language lateralization**.

**Study**	**Dextral n**	**LD (%)**	**AD (%)**	**Adextral n**	**LD (%)**	**RD (%)**	**LD diff (Dex-Adex)**
Arora et al., 2009	30	87	13	10	20	75	+67
Axmacher et al., 2009	24	83	17	10	40	60	+43
Badzakova-Trajkov et al., 2010	107	95	5	48	81	19	+14
Benson et al., 1999	11	100	0	8	75	25	+25
Berl et al., 2013	185	75	25	39	62	38	+13
Bethmann et al., 2007	26	92	8	5	50	50	+42
Binder et al., 1996	19	84	16	3	67	33	+17
Cai et al., 2008	10	90	10	9	44	56	+46
Chlebus et al., 2007	12	83	17	3	67	33	+16
Cobianchi and Giaquinto, 1998	18	94	06	18	61	39	+33
Deblaere et al., 2004	14	93	07	4	100	0	−7
Findlay et al., 2012	21	86	14	14	57	43	+29
Fleminger and Bunce, 1975	44	82	18	8	67	33	+15
Flöel et al., 2005	37	97	03	38	74	26	+23
Gaillard et al., 2007	80	80	20	20	40	60	+40
Groen et al., 2013	45	84	16	12	50	50	+34
Häberling et al., 2011	35	91	9	25	76	24	+15
Hirata et al., 2010	54	89	11	7	57	43	+32
Jansen et al., 2007	130	98	02	53	66	34	+22
Jones et al., 2011	47	92	02	16	56	36	+26
Khedr et al., 2002	25	84	16	25	68	32	+16
Knecht et al., 2000	155	95	05	132	78	22	+17
Krach et al., 2006	29	97	03	29	66	34	+31
Loring et al., 1990	91	80	20	12	75	25	+5
Mazoyer (sub)	144	94	06	153	84	16	+10
Niskanen et al., 2012	16	94	06	4	100	0	−6
Powell et al., 2012	42	100	0	40	80	20	+20
Pujol et al., 1999	50	98	02	50	82	18	+16
Spreer et al., 2002	18	78	22	5	40	60	+38
Springer et al., 1999/Szaflarski et al., 2002	50	78	22	50	78	22	0
Stewart et al., 2014	126	88	12	36	72	28	+16
Sveller et al., 2006	61	82	18	13	69	31	+13
Tzourio-Mazoyer et al., 2014	144	94	06	153	84	16	+10
Van der Kallen et al., 1998	14	100	0	6	17	83	+83
Vernooij et al., 2007	10	70	30	10	50	50	+20
Warrington and Pratt, 1973	52	98	02	23	78	22	+20
Westerhausen et al., 2006	42	81	19	47	66	34	+15
Whitehouse and Bishop, 2009	45	80	20	30	67	33	+13
Weighted mean		91%	9%		76%	24%	

*Classification techniques vary somewhat, as do criteria for bilateral language classification (hence my grouping of bilateral and right dominance classifications as anomalous dominance). The final column represents the difference between dextrals and adextrals in the percentage of that sample which is left hemisphere dominant. The weights in the associated meta analysis were used to calculated the weighted percentages at the bottom of the table. The difference between the two weighted LD means is equivalent to a risk ratio, dextrals to adextrals, of 1.17*.

A major concern, well understood by neuroimagers in this field, is the continuous nature of activation data revealed in individuals performing language-relevant tasks in the scanner. For our purposes here, we will ignore methodological differences (particularly those related to decisions regarding defining bilateral speech representation from continuous fMRI data—see Binder et al., 1996; Baciu et al., 2005; Bethmann et al., 2007; Vigneau et al., 2011; for some of the debates regarding precise procedures).

We also include studies in this analysis which use other measurement techniques based on blood flow, such as transcranial Doppler sonography (TDS). The grouping together of such diverse methods (ECT, fMRI, MEG, EEG, and TDS) may alarm researchers who use such techniques regularly. Nevertheless, in our mind they are less comfortably grouped with the aphasia literature, or with the indirect perceptual tasks such as DL or VHF experiments.

It is not surprising that the vast majority of studies examining the cerebral organization of language with these newer paradigms test right handers exclusively (e.g., Neville et al., 1998; Parker et al., 2005; Pillai and Zaca, 2011). On the other hand, some fMRI/EEG/TDS researchers may be biased by the overlap between dextrals and adextrals to the point that handedness is no longer even mentioned in the methods sections of individual papers (e.g., Wang et al., 2012; Bellugi et al., in press). This state of affairs is no doubt exacerbated by the rarity of adextrals in any small or medium sized sample of individuals, patient groups or otherwise.

Other fMRI experiments have contrasted reasonably large samples of dextral and adextral participants on various language, memory and spatial tasks, but the emphasis in analysis is on measures of central tendency from the entire group (e.g., Gur et al., 1982; Cuzzocreo et al., 2009) or they only report main effects or other data that do not allow for the risk ratio calculations used here (e.g., Miller et al., 2005). Structural investigations using techniques such as diffusion tensor imaging (DTI) are now appearing which include dextrals and adextrals as separate groups, but they often do not have functional data on their participants or, as is often the case, focus on measures of central tendency at the group level (Hagmann et al., 2006).

### Methods

Many of the papers included in this analysis were identified by related reference and cited reference searches for classic papers such as Rasmussen and Milner (1977). Literature searches for this set of studies on fMRI and handedness, TDS and handedness, ERP and handedness, included many papers that we then excluded for reasons above. We also identified papers which cite some of the original large n fMRI handedness studies including Benson et al. (1999), Knecht et al. (2000; a large n TDS paper), Pujol et al. (1999), Springer et al. (1999), etc. This final paradigm-based random effects meta analysis uses the data of Table 3 to create rate ratios for left dominance, dextrals relative to adextrals.

### Results

The 35 studies summarized included 1870 dextral and 1066 adextral participants. The results of this analysis appear in Figure 4. One unusual TDS paper (Basic et al., 2004) found 92% *right* brain dominance in their adextral group, a highly unusual result (equal to an odds ratio of 11.67 for this particular study, compared to a range of 1.00–2.08 for the other 34 experiments). Therefore, it was dropped from the analysis (Supplementary Material includes it for comparison purposes). The revised overall risk ratio for left hemispheric dominance in dextrals compared to adextrals is 1.21 (95% CI 1.15–1.28; *Q* = 56.34, *p* = 0.01, *I*^2^ = 41%). We also modeled % left dominance in these studies by weighting each group percentage by the meta-analytically derived inverse weighting. This procedure suggests a best estimate, on average, of left brain dominance in dextrals of 90% and in adextrals, 73% (data available on sheet 1 in Supplementary Material).

### Discussion

This overall effect estimate is remarkably similar to the one associated with Figure 3 based on the dichotic listening/visual half field results (overlap between these two meta analyses is less of an issue than overlap between the fMRI/ECT/TDS and WADA test analyses, discussed below in Experiment 6, although three studies do overlap with other paradigm specific meta analyses above).

## A meta meta analysis?

### Introduction

Recently, advocates of meta analytic techniques have pondered how subgroups can be compared statistically (Schmidt and Hunter, 2015). For example, the rate ratios for the different domains described here can be compared with one another, and the effects on heterogeneity can be modeled by including them all in an omnibus meta analysis. Adding or subtracting different subgroups (in a fashion not unlike hierarchical regression) could reveal informally the relative contributions to heterogeneity. One of the reviewers of a previous version of this manuscript suggested that all of the studies could be included in an omnibus meta analysis, with the degree of heterogeneity across the subgroups established.

According to the Cochrane collaboration (Deeks et al., 2011), such comparisons have to be made with caution. Differences in the magnitude of effects or degree of heterogeneity cannot be unambiguously related to subgroup membership exclusively. For example, in the particular case here, random effects meta analysis will re-weight all of the studies based on the inverse of their variance. This re-weighting means that within experiment sample sizes as well as the number of different experiments identified by the literature search will affect how different studies contribute. Nevertheless, we have performed an overall rate ratio meta analysis using all of the DL/VHF, WADA, and fMRI/ECT/TDS studies.

### Methods

An inverse variance random effects model instantiated in RevMan) 5.0 (2008), provided by the Cochrane Collaboration (http://www.cochrane.org/) was used for the omnibus analysis. It can be downloaded freely here: http://tech.cochrane.org/Revman. The graphical capabilities of this software are rather limited, so we have continued to use MetaXL for the main studies reported above. We used RefMan for this final analysis as it provides decent summary statistics about subgroups in a way that MetaXL does not. Identical rate ratios and confidence intervals are provided by both packages for DL/VHF, WADA tests, and fMRI/ECT/TDS analyses. The Cochrane Handbook recommends random effects for subgroup analysis: “Tests for subgroup differences based on random-effects models may be regarded as preferable to those based on fixed-effect models, due to the high risk of false-positive results when comparing subgroups in a fixed-effect model (Higgins and Thompson, 2004)” (Deeks et al., 2011; 9.6.3.1).

### Results

Supplementary Material provides the graphical summary, rate ratios and heterogeneity estimates for the entire analysis as well as the subgroups. Note that the weights applied to each individual study change relative to those computed when each subgroup was subjected to its own meta analysis (Figures 2–4). In fact, the sheer number of DL/VHF tests, along with their relatively large numbers of dextral and adextral participants, means that they account for 59.3% of the overall analysis (WADA = 16.5% and fMRI/ECT/TDS = 24.1%). Unsurprisingly, the overall rate ratio estimate is quite similar to those for the DL/VHF and the fMRI/ECT/TDS analyses: 1.25 (95% *CI* = 1.22, 1.29).

RevMan uses the significance test for subgroup differences recommended by Borenstein et al. (2008). Essentially it tests for heterogeneity across subgroups rather than across individual studies. It also provides an I^2^ estimate describing variability due to subgroup differences that is not accounted for by sampling error. We have violated the assumption that the datasets are truly independent, as some participants from WADA tests were also scanned in parallel fMRI/MEG experiments (e.g., Spreer et al., 2002; Axmacher et al., 2009; Hirata et al., 2010). In this instance, the subgroup value of Chi^2^(2) = 6.69, *p* < 0.05. *I*^2^ = 70.1%, suggesting significant variability across subgroups. This significant heterogeneity may be largely due to the WADA test subgroup, as an additional “semi” omnibus test including only the 35 fMRI/ECT/TDS experiments and the 72 DL/VHF experiments reveals no significant heterogeneity (Chi^2^(1) = 0.07, *p* > 0.05) and a significant rate ratio (*Z* = 13.67, *p* < 0.0001) of 1.22 (95%C.I. = 1.19, 1.26).

### Discussion

As noted above, comparing these subgroups has to be done with caution, as there are considerable differences in study number, within study sample size and some non-orthogonality, as some individuals appear in more than one paradigm. Nevertheless, this final analysis does suggest heterogeneity across methods, at least when comparing the WADA test analyses to the others. The analysis supports what the individual meta analyses above suggest—little difference in rate ratios across DL/VHF experiments and the (mainly) more recent studies using fMRI/ECT/TDS methods. There is little overlap of participants between these two sets of experiments. These data are somewhat surprising, given the indirect nature of these behavioral tests and how they may be confounded with attentional and perceptual factors. (Such caveats are rarely made about the results from the newer methods such as fMRI.)

## General discussion

All of the analyses, bar one, show increased left brain dominance for language in the dextrals of approximately 20%. The least conclusive analysis, in terms of the absolute difference between dextrals and adextrals was for aphasia incidence after left brain damage. Although an effort was made to exclude patient series where some pre-selection was made or implied, it is difficult to evaluate the success of such an enterprise. For example, most of those studies were published pre-1980, which means that, for obvious reasons, additional information about how the experiments were conducted is no longer possible to come by. The most recent, Kimura (1983), for example, parallels her arguments from the 1960s for no difference between dextrals and adextral samples on dichotic listening: she found no differences between her adextrals and dextrals in aphasia incidence after right brain damage (3 vs. 2%, respectively). This sample did show, however, that left brain damage was less likely to lead to aphasia in the adextrals (23% to the dextral 41%). This pattern of data is slightly counterintuitive to our first two meta-analyses, which suggest that aphasia after *right* brain damage separates dextrals and adextrals more effectively.

Is Kimura's sample unusual in some respect? The number of adextrals reported was amongst the better in this kind of study (37 with left brain damage, 30 with right brain damage). She claims that 9% of the sample of patients with unilateral brain damage were adextral (a sensible estimate given what is known about handedness); although her definition of adextrality was quite inclusive (if <7/8 items on her handedness questionnaire indicated the right hand). This kind of in depth analysis of individual papers is dangerous in this context, of course, as scientists tend to be overly analytic of results that are counterintuitive (see below).

The evidence for dysphasia incidence after right brain damage is clearer. The susceptibility of adextrals to dysphasia is over six times higher relative to the dextral samples. In spite of this clear difference, there still remains some uncertainty about whether or not all dextrals and adextrals would have been tested routinely for aphasia after right brain damage. Sadly, these sorts of studies have largely gone out of fashion, in spite of the fact that stroke registers, computerized databases and so forth should mean that these kinds of data could be collated after the fact, in many centers, at a time where much more information about etiology, lesion size and location, could be recorded routinely as part of the electronic record. Handwriting hand, in a pinch, would suffice, if sample sizes were sufficiently large (many handedness researchers may have concerns about such a recommendation). Almost all of the relevant information (in the later twentieth century) related to atypical dominance, lesion location and so on has come from the single case literature on crossed aphasias, apraxias and hemispatial neglect. We argue that the limitations of WADA testing of people with epilepsy are circumvented with the study of patients with acute brain damage.

Aphasia data aside, the other meta analyses differ slightly in terms of the precise rate ratios obtained. The rate ratio from WADA testing (1.36) is somewhat higher than that obtained from the DL/VHF and fMRI/ECT/TDS analyses (1.22 and 1.26, respectively). As Table 2 shows, language dominance is driven away from the hemisphere of epileptic focus to some extent in both dextral and adextral patients. These issues are discussed in great detail in several analyses (Helmstaedter et al., 1997; Springer et al., 1999; Dijkstra and Ferrier, 2013; Stewart et al., 2014).

In spite of the larger rate ratio obtained from the WADA experiments, the similarity in rate ratios obtained from DL/VHF and fMRI/ECT/TDS is encouraging. Heterogeneity is clearly an issue within both domains (*I*^2^ = 28.7% for DL/VHF, 62.4% for fMRI/ECT/TDS), but our original suggestion that an inclusive meta analytic approach could cope with some of this heterogeneity is supported by the consistent rate ratios. The convergence from these different domains is noteworthy, given that many neuroimagers are struck by bilateral activations in any language-related task. Cerebral specialization has received less attention in the last 20 years than expected, given its huge importance in the earliest type of “cognitive neuroscience”—neuropsychology.

A rate ratio cannot be used to predict the percentage of dextrals or adextrals who are likely to be left hemisphere dominant for speech. One could use the ratio to predict that value in one group, if the other mean percentage is known or hypothesized. Instead of that kind of calculation, we used the inverse variance weights assigned to each experiment to estimate weighted dominance percentages. The results are interesting, but may need more careful modeling. For DL/VHF, the weighted estimate is 83% left brain dominance in the dextrals; 68% left brain dominance in the adextrals (a 15% difference in the expected direction). For WADA our estimates suggest 87% left dominance in the dextrals and 65% left dominance in the adextrals a 22% difference). Finally, for fMRI/ECT/TDS, the numbers are 88% left dominance in the dextrals and 64% left dominance in the adextrals (a 24% difference). Are there any good theoretical or empirical reasons to place more stock in one of these estimates more than the others?

These estimates, for the dextrals in particular, are slightly lower than the 90%+ predicted by many of the early group studies (e.g., Rasmussen and Milner, 1977). In addition, genetic models such as McManus' DC theory (McManus, 1985) and Annett's Right Shift theory (2000) make similar >90% left dominance predictions for dextrals. Of course any estimates will depend to some extent on how liberal or conservative the criterion is for inclusion in a left brain dominant or no dominance group (grouped with right brain dominance for our purposes here). For meta analyses and the associated rate ratios, what mattered was that within-study the same criterion was applied to dextral and adextral groups. These estimates for left brain dominance would change with criteria: for example in the work of Brysbaert, Van Der Hagen and their colleagues, a conservative criterion was adopted to ensure strong hemispheric asymmetry in a number of identified individuals. That criteria results in estimates of no atypical dominance in dextrals and about 10% in adextrals (Brysbaert et al., 2012; Van der Haegen et al., 2013a,b) well below the estimates derived in the present analysis. It may be that our estimates of left brain dominance of 85% in dextrals may be somewhat conservative, by assigning more weak “left hemispheric” scores on tasks such as dichotic listening to a no dominance grouping.

Each of these research domains has its own associated weaknesses and strengths for helping to determine the underlying distributions of cerebral asymmetry. WADA testing, as noted above and elsewhere, is limited methodologically for several reasons (what counts as speech arrest, test-retest reliability, etc.), but the most concerning limitation is that congenital brain damage may bias dominance in some unknown (and unknowable) proportion of the patients (see Table 2). A strength of WADA, however, is the relatively unambiguous trichotomous data that it provides.

These estimates are in stark contrast to those from neuroimaging, where several methodological issues make simple left, right, bilateral classifications more contentious. For example, calculating a laterality index from functional data requires some hard decisions about regions of interest and thresholding (e.g., Jansen et al., 2006; Abbott et al., 2010), equating regions from each hemisphere which are not structurally identical (Shapleske et al., 1999), and the nature of baseline conditions (Seghier, 2008). Practical issues for imaging include expense (these asymmetry studies benefit from large sample sizes) and the difficulties inherent with interpreting data from single participants (Bosch, 2000; Fedorenko et al., 2010).

Sample size and expense are not particularly crucial issues for DL/VHF studies with neurotypical university undergraduates. These methods, as discussed above, are the most indirect measures of brain asymmetry, have relatively poor test-retest reliabilities and estimates in single participants can be seriously distorted/biased by attentional strategies, task demand and the like.

A reviewer of a previous draft suggested rating studies for their quality (i.e., presence of absence of the different cofounding effects mentioned above, for example) in order to evaluate the sources of heterogeneity more carefully. This suggestion is indeed tempting, as several of the estimates in each domain strike us as improbable, but were included nevertheless (with one exception in the fMRI/ECT/TDS paradigm analysis). In fact, after generating each forest plot it is extremely tempting to discard the wilder appearing estimates which appear outside the range of the other studies. In the ideal world of “new statistics,” file drawer problems and biases against null effects and the like would be minimal, as ideally all datasets would be available electronically for meta analytic use (Cumming, 2012, 2013). We do not as of yet operate in such a world. Impressions about quality inevitably will reflect some of the personal biases about what the “true” differences between dextral and adextrals are. Another difficulty with a quality approach is that for many of the possible sources of noise discussed above and in detail elsewhere, their presence, absence or magnitude is hard to quantify. In some cases (as suggested by Kimura, 1983 regarding the aphasia incidence literature), there are reasons to suspect whether or not samples are truly random, or that all dextrals and adextrals were tested and none were preselected in any fashion whatsoever. In a few instances, the samples were not selected for writing hand alone. We have largely ignored historical covariates like familial sinistrality, foot preference or sex, as these tended to apply to both dextral and adextral samples in a similar fashion (in so far as we could tell). Nevertheless, it's likely they would muddy the waters somewhat, if the focus was restricted to a small number of key experiments.

### Theoretical significance of proportions of dextral and adextral language dominance

Excessive concern over precise estimates of cerebral dominance in adextrals, relative to dextrals, might seem a rather specialist sort of worry. Obviously, if the proportion of left hemisphere dominance in adextrals is much higher than these meta analyses suggest, then adextrals and dextrals may not differ in this aspect. Such a result would remove at least one of the major sources of neuropsychological interest in handedness. On the other hand, if adextrals (or very strong left handers at least; see Knecht et al., 2000) were largely right hemisphere dominant for speech, the so-called “Broca's rule” would actually apply, therefore much of the mystery surrounding left handers would largely disappear (i.e., handedness and cerebral dominance for speech and language would predict one another in some direct fashion). The present data, in spite of some of the limitations discussed above, suggest a more complex relationship between handedness and cerebral specialization than either of those two extremes. Practically speaking, a more precise estimate of the degree of language dominance, in both dextrals and adextrals, does have important ramifications, in at least three ways.

First, identifying the more appropriate “phenotype” for many studies (behavioral, genetic, neuroimaging, EEG etc.) could be aided considerably by knowing how many (and which) adextrals have crossed or uncrossed control of speech and limb function. For example, studies of asymmetries that tend to favor the right hemisphere in dextrals, such as face processing (Kanwisher, 1997; Yovel et al., 2008; Meng et al., 2012) and in particular, functions related to paralinguistic aspects of speech such as prosody (van Rijn et al., 2005; Ross and Monnot, 2008) would benefit greatly from knowing which individuals are largely left or right hemisphere dominant for typical speech and language function. In fact, an older literature on “complementary hemispheric specialization” (Bryden et al., 1983a; Elias et al., 1999a,b) has been largely forgotten about, but is ripe for a revival (Cai et al., 2013).

Similarly, organization of subregions of left hemisphere networks in individual or groups of dextrals (Fedorenko et al., 2012a,b) could be contrasted with their counterparts in right dominant individuals, if they could be identified at the individual or small group level. Additionally studies of increased incidence of adextrality in conditions such as dyslexia, autism, developmental coordination disorder and language-specific impairment might be more conclusive if cerebral control of hand and speech were the independent variables, rather than handedness (typically restricted to writing hand).

Second, more precise estimates of language dominance proportions could open up new studies of manual behaviors, particularly ones which are *not* hand-writing. It may be that one of more of these other manual behaviors (e.g., reaching and grasping, for example; Gonzalez et al., 2007; Gonzalez and Goodale, 2009) or gesturing (Kimura, 1973a,b) might be right hand biased in a significant proportion of “left” handers (as defined by writing hand). In other words an ideal predictor of cerebral dominance for language would be right hand biased in approximately 85% of a sample of dextrals, and 65% of a sample of adextrals. Sadly, almost all studies which contrast dextrals and adextrals on measures of hand choice, hand preference patterns, indirect measures of asymmetry such as DL and VHF studies, and so on inevitably report measures of group tendency and variability and fail to say anything about subgroups (of particular relevance in the adextral samples if sufficiently large and well characterized). Our suspicion is that these possible predictor behaviors need to be measured in the lab or the real world, rather than reported on via a paper and pencil questionnaire (Carey et al., 2009; Gonzalez and Goodale, 2009).

For example, the relatively poor correlations between VHF and DL experiments, or test-rest correlations with the same measures might benefit from a more considered analysis of proportions of the samples who show effects in one direction or the other. A test might have poor reliability because its precise estimate is noisy, yet it might classify individuals dichotomously quite well. It seems probable that people with larger scores on these indirect tests might be less likely to show significant changes on retest (at least in direction), in which case participant performance could be examined more carefully in the individuals who score nearer to zero (are they following task instructions, are they in fact less lateralized across many measures etc.). This kind of approach presupposes a more considered analysis of an individual's performance on two versions of the same test or across different indirect tests.

A third reason why precise estimates of language dominance are of interest is related to sensorimotor control and handedness. In the vast majority of dextrals, the hemisphere more specialized for speech and language is largely in control of the dominant hand, at least at the levels of motor/premotor output and somatosensory input. From a handedness perspective, clearly something very different is going on in the majority of adextrals. In this context, (related to, but not synonymous with, motor theories of speech perception; e.g., Lieberman, 2006; MacNeilage, 2008), there should be subtle benefits (“privileged access”) in sensorimotor control for having the dominant hand intimately interconnected with the motor, premotor and somatosensory cortices of the same hemisphere that largely controls the speech musculature (Goodale, 1988; Carey and Otto-de Haart, 2001). A corollary of this idea is that, for the *majority* of adextrals, the non-dominant hand might enjoy benefits for the same reason, at least when compared to the non-dominant hand of dextrals, which statistically, is likely to have privileged access to attentional and visuospatial networks (Mieschke et al., 2001; Carey and Liddle, 2013). Surprisingly, very few studies compare absolute levels of performance in these “four hands” (the few that do are typically a little underpowered when it comes to the size of the adextral sample; e.g., Goodale, 1990; Boulinguez et al., 2001).

In conclusion, efforts to establish precise estimates for dextral and adextral language asymmetry are challenged by pre-selection biases, poor sample sizes, and incomplete reporting of data. The tendency for adextrals to be left hemisphere dominant is conceived (by different scientists) to be an unwanted source of heterogeneity (they therefore just test dextrals). Another approach is to ignore adextrals altogether (e.g., don't record handedness at all, or at least don't report it), as they are relatively rare folk who, for the most part, are arranged as the right handed majority anyway. Nevertheless, these meta analyses reinforce the idea that adextrals have an unusual cerebral arrangement vis-à-vis the control of speech and language vs. control of their dominant hand.

### Conflict of interest statement

The authors declare that the research was conducted in the absence of any commercial or financial relationships that could be construed as a potential conflict of interest.

## Footnotes

^1^We have used the term “anomalous dominance” here for right brain dominance, bilateral or no dominance categories in any particular study. The term is not intended to presuppose that non-left dominance is somehow pathological, as implied by the “Geschwind-Behan-Galaburda” model of the 1970s (see Bryden et al., 1994; Dellatolas, 1994, for detailed discussion).

^2^The precise numbers of dextral and adextral patients screened after right brain damage were not available in Luria (1970), hence its exclusion from the second but not the first analysis.

^3^For reviews of neural plasticity as a function of age, see Dennis (2000). Early left hemispherectomy often results in right hemispheric language dominance, which in many ways resembles the typical functional organization of language in the left hemisphere (Staudt et al., 2002).

^4^Familial sinistrality was once considered an extremely important moderator of cerebral asymmetries (e.g., Hécaen and Sauguet, 1971; reviewed by Bishop, 1980) but has largely fallen out of favor (e.g., Orsini et al., 1985; Bishop, 1990; although see Willems et al., 2014, which argues for its reinclusion as a moderator variable).

^5^A related literature on concurrent hand movements and speech will not be referred to here, as the vast majority of these papers only report measures of central tendency (e.g. Lomas and Kimura, 1976; Sussman, 1982; Murphy and Peters, 1994).

^6^Several studies which focussed on dextrals exclusively will not be summarized here. Representative papers that result in cerebral asymmetry classifications largely consistent with the dextral means from fMRI, WADA, ECT and TDS include (Kompus et al., 2012; Ocklenburg et al., 2013).

^7^We are also excluding studies where inclusion criteria influenced the likelihood of increased incidence of anomalous dominance (e.g., Sabbah et al., 2003), with the exception of the pursuit of adextrals of course.
