# Supplementary figures and images for: Quantifying cerebral asymmetries for language in dextrals and adextrals with random-effects meta analysis
Source: Front Psychol. 2014 Nov 4;5:1128. doi: 10.3389/fpsyg.2014.01128 (PMC4219560; doi:10.3389/fpsyg.2014.01128)

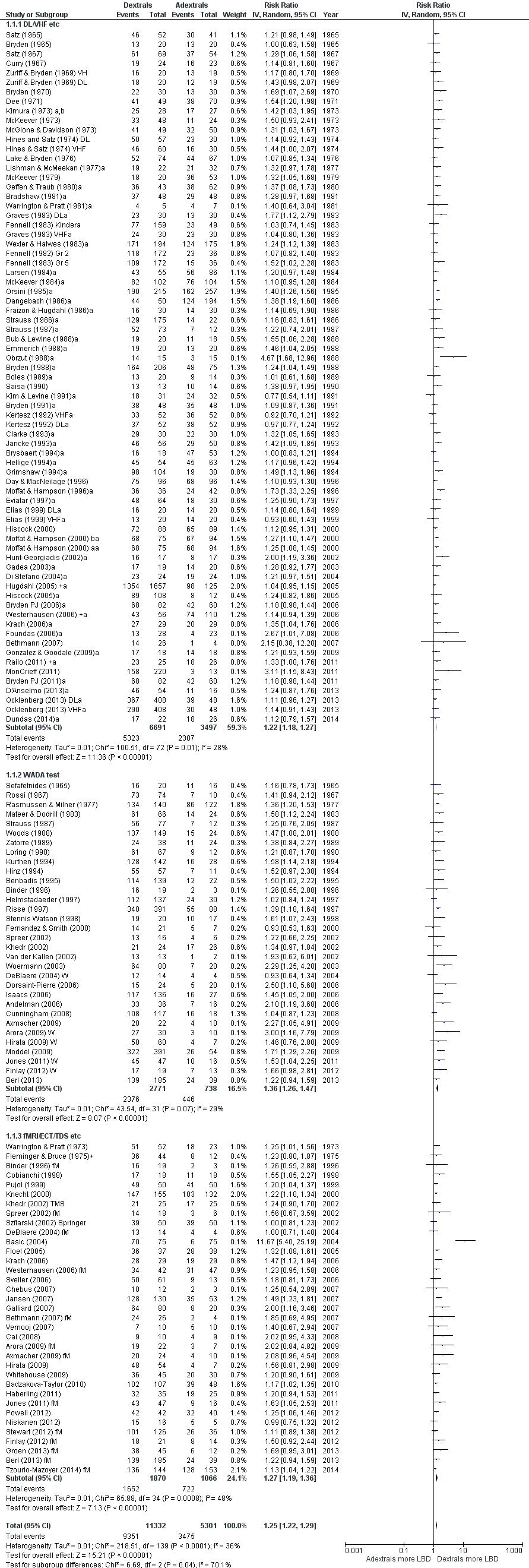

Supplement: Supplementary file 6 [file Image6.JPEG]
